# Magnetic resonance angiography and transcranial Doppler ultrasound findings in patients with a clinical diagnosis of vertebrobasilar insufficiency

**DOI:** 10.6061/clinics/2020/e1212

**Published:** 2020-01-14

**Authors:** Arlindo Cardoso Lima Neto, Edson Bor-Seng-Shu, Marcelo de Lima Oliveira, Alberto Macedo-Soares, Flávia Renata Topciu, Roseli Saraiva Moreira Bittar

**Affiliations:** I Departamento de Otoneurologia, Faculdade de Medicina FMUSP, Universidade de Sao Paulo, Sao Paulo, SP, BR; II Departamento de Neurologia, Faculdade de Medicina FMUSP, Universidade de Sao Paulo, Sao Paulo, SP, BR; III Departamento de Geriatria, Centro Universitario Lusiada (UNILUS), Santos, SP, BR; IV Departamento de Geriatria, Centro Universitario Sao Camilo, Sao Paulo, SP, BR

**Keywords:** Vascular Vertigo, Arteriography, Transcranial Doppler Ultrasound, Magnetic Resonance Angiography, Diagnosis, Vertebrobasilar Insufficiency

## Abstract

**OBJECTIVE:**

To evaluate the findings of magnetic resonance angiography (MRA) and transcranial Doppler ultrasound (TCD) in patients with a clinical diagnosis of vertebrobasilar insufficiency (VBI).

**METHOD:**

From our outpatient neurotology clinic, we selected patients (using the criteria proposed by Grad and Baloh) with a clinical diagnosis of VBI. We excluded patients with any definite cause for vestibular symptoms, a noncontrolled metabolic disease or any contraindication to MRA or TCD. The patients in the study group were sex- and age-matched with subjects who did not have vestibular symptoms (control group). Our final group of patients included 24 patients (study, n=12; control, n=12).

**RESULTS:**

The MRA results did not demonstrate significant differences in the findings between our study and control groups. TCD demonstrated that the systolic pulse velocity of the right middle cerebral artery, end diastolic velocity of the basilar artery, pulsatility index (PI) of the left middle cerebral artery, PI of the right middle cerebral artery, and PI of the basilar artery were significantly higher in the study group than in the control group, suggesting abnormalities affecting the microcirculation of patients with a clinical diagnosis of VBI compared with controls.

**CONCLUSION:**

MRA failed to reveal abnormalities in patients with a clinical diagnosis of VBI compared with controls. The PI of the basilar artery, measured using TCD, demonstrated high sensitivity (91%) and specificity (91%) for detecting clinically diagnosed VBI.

## INTRODUCTION

“Vertebrobasilar insufficiency” (VBI) is defined as transitory ischemia of the vertebrobasilar circulation, which supplies the medulla, cerebellum, pons, midbrain, thalamus, and occipital cortex (1,2). Vertigo and dizziness are the most frequent clinical symptoms of VBI and may quite often occur in isolation, without any additional symptoms. Other frequent symptoms include diplopia, visual blurring, and focal neurological deficits. Those symptoms occur following acute episodes of focal ischemia and, by definition, disappear within 24 hours without further sequelae (3,4). Although underdiagnosed, VBI is a frequent cause of dizziness, especially in elderly patients: up to 5% of all individuals over the age of 60 years presenting with dizziness have a subjacent VBI (5-7). Nonetheless, it is imperative to accurately diagnose those transitory ischemic events, considering that studies have demonstrated a significant positive correlation between those episodes of transitory ischemia and the odds of a future stroke (1,8-11).

The diagnosis of VBI is clinical, but imaging tests may be used to confirm the disease. The gold-standard test for VBI is arteriography (or angiography) (12), which is an invasive and risky test, especially in elderly patients; thus, the risks of arteriography may outweigh the potential benefits.

In this study, we aimed to evaluate abnormalities in the magnetic resonance angiography (MRA) and transcranial Doppler ultrasound (TCD) findings in patients with a clinical diagnosis of VBI.

## MATERIALS AND METHODS

### Patient selection

Using a nonprobability, purposive sampling technique, we selected patients from the neurotology outpatient clinic of the “Hospital de Clínicas” of the “Faculdade de Medicina da Universidade de São Paulo (HC-FMUSP)” who had a clinical diagnosis of VBI. Patients were selected using the clinical characterization of VBI proposed by Grad and Baloh (3) (patients with vertigo associated with one or more of the following symptoms: visual dysfunction; drop attacks; unsteadiness/incoordination; extremity weakness; confusion; headache; hearing loss; loss of consciousness; extremity numbness; dysarthria; tinnitus; and perioral numbness). In our clinic, all patients who have dizziness are routinely subjected to an extensive clinical investigation protocol, which includes anamnesis, clinical and otolaryngological examinations, testing of all cranial nerves, evaluation of the static and dynamic balance (Romberg and Fukuda stepping tests), coordination tests (index-nose, index-index and diadochokinesis); tonal and vocal audiometry; tympanometry; complete oculographic examination; and caloric tests. All subjects voluntarily agreed to participate in the research and signed informed consent forms.

We included patients based on the following inclusion criteria: (1) continuous or recurrent dizziness, lasting more than 12 weeks, plus one or more of the following symptoms: visual complaints (including blurry vision and diplopia), drop attacks, dysphagia, dysarthria, facial or limb extremity paresthesia, mental confusion, loss of consciousness, or headache; and (2) one or more risk factors for vascular diseases (including high blood pressure, controlled diabetes, smoking, dyslipidemia, obesity, heart diseases, and peripheral artery disease). The exclusion criteria were defined as follows: (1) dizziness secondary to any other definite etiology; (2) noncontrolled metabolic or autoimmune disease; (3) definite migraine (13); or (4) any recommendation against or physical limitation to any of the diagnostic tests we used in this study.

To better assess (and exclude) the presence of possible associated comorbidities or other factors that may be the underlying cause of the vestibular symptoms, all participants were subjected to a geriatric cardiovascular evaluation, which was conducted by a specialist physician from the geriatrics department. During the clinical evaluation, the patients underwent complete clinical and physical examinations, and the blood pressure was measured in the supine decubitus, sitting and standing positions. Additionally, patients underwent blood testing, including full blood cell count, glycated glucose hemoglobin, lipid profile, thyroid function (thyroid-stimulation hormone and thyroxine), and renal (creatinine and blood urea nitrogen) and liver (aspartate transaminase and alanine transaminase) function tests. The physician from the geriatrics department also requested additional tests (electrocardiography, Holter, and tilt tests) to exclude potential causes for the vestibular symptoms other than VBI. Although our initial study protocol included angiography as a diagnostic test, most of the patients in our study did not consent to the examination after we disclosed the potential risks. Furthermore, some of the patients had several comorbidities that could constitute additional risks for performing the tests; therefore, we (and our Ethics in Research Committee) considered that performing angiography in disregard of those risks would be ethically questionable. Thus, the patients were diagnosed using clinical criteria.

Using a nonprobability, convenience sampling method, we also selected patients from the geriatric outpatient clinic as controls; these patients were age- and sex-matched to those in our study group. We defined controls as patients who did not have (1) dizziness, (2) a recent history of vascular disease, or (3) migraine or (4) had any recommendation against or physical limitation to any of the diagnostic tests in this study. The first author of this study selected and performed the clinical evaluation of the patients in both groups. An experienced neuroradiologist was responsible for performing MRA and TCD for patients in both groups.

Our final study group included twelve patients who had a clinical diagnosis of VBI (four men and eight women). Their mean age was 72.66 (median=72.66; standard deviation (SD)=8.35). The control group included 12 patients who were sex- and age-matched to the patients in the study group.

### Ethical considerations

The Ethics in Research Committee of the HC-FMUSP approved this study (0016/10). Our methodology also complies with the principles dictated by the Declaration of Helsinki.

### MRA

The intracranial MRA was performed using a 1.5-Tesla machine and the three-dimensional angiographic sequence with the “time-of-flight” (3D-TOF) inflow technique in the axial plane without intravenous contrast. The area of acquisition extended from the foramen magnum to the cranial vertex. The 3D-TOF technique is a validated method for evaluating the intracranial branches of the carotid artery; it provides better resolution and more precise images than T1-weighted MRI technique with intravenous contrast infusion; thus, the 3D-TOF technique is recommended as the best option for evaluating intracranial vessels (11).

Cervical MRA was performed using a 1.5-tesla machine and the three-dimensional angiographic gradient-echo (3D-GR) sequence in the coronal plane, with a short repetition time (RT) and echo time (ET). During this test, we injected a gadolinium solution of 0.15 mmol/kg at an infusion rate of 2 ml/sec, followed by 20 ml of saline solution. The acquisition area extended from the aortic arch to the cranial base.

We evaluated the right and left internal carotid arteries, basilar arteries and the V1, V2, V3 and V4 branches of the right and left vertebral arteries. When present, stenosis in each segment was graded as follows: (1) no stenosis; (2) less than 50% of stenosis; (3) 50-69% of stenosis; and (4) more than 70% of stenosis.

Only one radiologist was responsible for analyzing the images. The analyses were made using the original images and multiplanar reconstructions with volume rendering (VR) and maximum intensity projection (MIP).

### TCD

We performed TCD to evaluate the possible pathological findings in patients with VBI. TCD was performed as per the method described by Aaslid et al. in 1982 (10). We used the EZ DOP/DWL Compumedics ultrasound system, which had a 2-MHz pulsatile wave emission transducer.

A neurologist with expertise in ultrasound performed all the TCD tests. Using TCD, we evaluated the middle cerebral arteries, the intracranial branches of the carotid arteries, the superior extracranial (V3) and intracranial (V4) portions of the vertebral arteries, and the basilar arteries. The test was performed using the temporal, orbicular, submandibular, and suboccipital windows. Additionally, we performed Doppler ultrasound examination of the carotid arteries and the V1 and V2 segments of the vertebral arteries; in cases where the blood flow in the vertebral arteries was abnormal, the subclavian arteries were analyzed as well.

We considered the following continuous quantitative variables: the peak systolic velocity (PSV), end diastolic velocity (EDV), and pulsatility index (PI). Based on the systolic and diastolic blood velocity obtained by the TCD flow spectrum, the mean velocity (MV) of the blood flow and the PI were automatically calculated by the equipment. If the ultrasound signal was attenuated, the calculations were performed manually using the following formulas: mean velocity = (peak systolic velocity - end diastolic velocity) + peak systolic peak velocity / 3; and PI = (peak systolic velocity - end diastolic velocity) / MV. Thus, the PI constitutes a ratio between the systolic and diastolic blood flow velocity , with a larger PI indicating increased resistance to blood flow in the microcirculation (14).

### Statistical analysis

To compare the results between the two groups, we used the nonparametric Mann-Whitney test. To establish the cutoff point for each significant variable, we created receiver operating characteristic (ROC) curves. Results were considered statistically significant when the *p*-value was less than 0.05.

## RESULTS

Regarding the MRA results, we did not observe significant differences in the degree of obstruction in the internal carotid arteries, basilar arteries or four segments of the vertebral arteries between the study and control groups (Table 1). Furthermore, we did not observe significant narrowing or obstruction of the arteries of any volunteer. We found similar patterns in the distribution of areas with white matter hyperintensity (suggesting microangiopathy) in the cerebral parenchyma of patients in both groups. Other structural abnormalities observed were as follows: (1) one patient from the study group had a bilateral fetal origin of the posterior communicating arteries; (2) two patients (one from each group) had a hypoplastic right vertebral artery; and (3) one patient from the control group had a persistent left trigeminal artery (Supplementary Material).

TCD revealed a significant difference in several tested hemodynamic parameters between the study and control groups, as follows: the PI of the left and right middle cerebral arteries; the PI of the basilar artery; the PSV of the right middle cerebral artery; and the EDV of the basilar artery (Table 2). We did not observe any significant abnormalities in the Doppler ultrasound findings of the vertebral or carotid arteries. No significant variations in blood flow through the arteries were observed during changes in the head position.

### ROC curves and analysis of the significant variables

#### PI of the left middle cerebral artery

**Figure 1:**
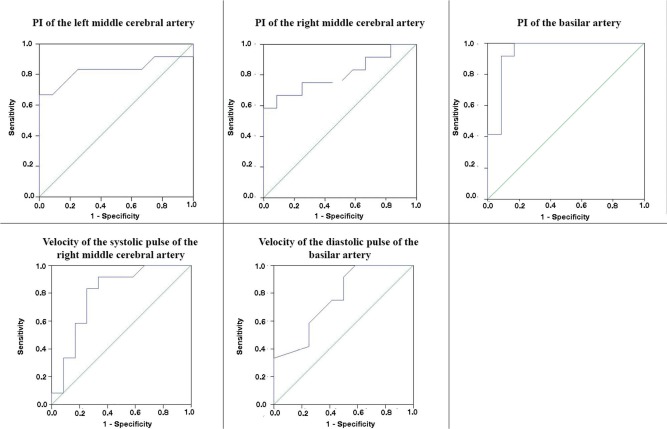
ROC curves of the PI of the left and right middle cerebral arteries and the basilar artery, the peak systolic velocity of the right middle cerebral artery, and the end diastolic velocity of the basilar artery.

The ROC curve of the PI of the left middle cerebral artery showed that higher sensitivity and specificity values were obtained when the test result was higher than 0.85 (Figure 1).

Considering the cutoff of 0.85, the sensitivity of the PI of the left middle artery was 83.3%, the specificity was 75%, the positive predictive value (PPV) was 76.9%, and the negative predictive value (NPV) was 81.8% in diagnosing VBI.

#### PI of the right middle cerebral artery

The ROC curve of the PI of the right middle cerebral artery showed that higher sensitivity and specificity values were obtained when the test result was higher than 0.88 (Figure 1).

Considering 0.88 as the cutoff, the sensitivity, specificity, PPV, and NPV of the PI of the right middle artery were all 75% in diagnosing VBI.

#### PI of the basilar artery

The ROC curve of the PI of the basilar artery showed that higher sensitivity and specificity values were obtained when the test result was higher than 1.01 (Figure 1).

Considering 1.01 as the cutoff, the sensitivity, specificity, PPV, and NPV of the PI of the basilar artery in clinically diagnosing VBI were all 96.1%.

#### PSV of the right middle cerebral artery

The ROC curve of the PSV of the right middle cerebral artery showed the highest sensitivity and specificity values when the test result was higher than 76.00 (Figure 1).

Considering 76.00 as the cutoff, the sensitivity of the PSV of the right middle cerebral artery was 83.3%, the specificity was 75%, the PPV was 76.9%, and the NPV was 81.8% in diagnosing VBI clinically.

#### EDV of the basilar artery

The ROC curve of the EDV of the basilar artery showed that higher sensitivity and specificity values were obtained when the test result was higher than 24.00 (Figure 1).

Considering 24.00 as the cutoff, the sensitivity, specificity, PPV, and NPV of the EDV of the basilar artery were all 66.6% in diagnosing VBI clinically.

## DISCUSSION

The sensitivity and specificity of arteriography in detecting abnormalities affecting the posterior circulation are higher than 90%; thus, it is considered the gold-standard examination for diagnosing VBI (12,15,16). However, there are inherent risks to arteriography, including bruising, arterial occlusion, thromboembolism, retroperitoneal hematoma, allergic reactions, stroke, neurological damage, and death (11,16,17). Subjecting patients who have minor symptoms to a risky diagnostic test may give rise to ethical and legal issues. In addition, the clinical diagnostic criteria for vestibular symptoms of vascular origin are well described in the literature (1-3,6); therefore, arteriography should be indicated only in specific cases (6).

In our study, we performed a thorough and extensive clinical evaluation of patients with suspected VBI to exclude patients with any other underlying cause for the vestibular symptoms. Although the strict selection criteria were applied with the aim of eliminating possible biases, they limited the number of subjects in the study group, especially considering that dizziness of vascular origin mostly affects older patients, who may have chronic, metabolic, and degenerative diseases. However, we are confident that our final study group comprised patients who strictly complied with the guidelines for diagnosing dizziness of vascular origin (1-3,6). Thus, after carefully considering each of those issues, we aimed to evaluate the value of two less invasive tests (MRA and TCD) in detecting intracranial vascular abnormalities in patients with a clinical diagnosis of VBI.

Regarding MRA, our results failed to demonstrate significant differences between the study and control groups, suggesting that MRA may not be a reliable diagnostic test in patients with a clinical diagnosis of VBI. Our results are similar to those described by Grad and Baloh (3), who failed to demonstrate a difference in the MRA findings of patients with a clinical diagnosis of VBI but who had abnormal angiograms - the authors hypothesized that MRA is not a reliable test in cases of transient ischemia without infarction. On the other hand, Nakagawa et al. (18) demonstrated significant MRA findings in patients with VBI compared with controls. However, the authors used a scoring system to describe their findings, and therefore, we believe that the authors may have included in their results nonsignificant obstructions affecting more than one segment. To decrease the risks of including such nonsignificant obstructions, we evaluated and compared the presence of an obstruction in each arterial segment individually in patients with VBI compared with controls.

MRA demonstrated signs of microangiopathy (gliosis, white matter hyperintensity) in all patients in the study and control groups. These results suggest that those signs of microangiopathy may not reflect abnormalities associated with VBI, as they may be associated with age and other coexisting diseases (arterial hypertension, dyslipidemia, diabetes), which affect the distal vessels of elderly patients. MRA can also indicate an apparent absence of blood flow in vessels with turbulent blood flow (smaller or partially obstructed vessels). Therefore, the lack of a difference in the MRA findings between our groups could have occurred due to false-positive results in the control group.

We found significant differences in several TCD variables, including the PI of the basilar and middle cerebral arteries and the systolic and diastolic velocity of the middle cerebral and basilar arteries. We found a decreased EDV of the basilar artery in patients with VBI; consequently, the PI in the study group was higher than that in the control group. The cutoff (calculated using the ROC curve) of the PI in the group of patients with VBI was within the superior normality levels calculated by Barbosa et al. (19). Considering that Arnolds and Reuterns (14) reported that the PI is directly related to the resistance to the blood flow in the microcirculation, our results suggest that the increased PI in our study group indicates that a higher resistance to the blood flow occurs in the terminal circulation, rather than in great vessels. We may infer that the most critical pathological changes leading to dizziness of vascular origin occur in the microcirculation of the brain, as demonstrated by an indirect index - the PI; these results may indicate that patients with a clinical diagnosis of VBI have diffuse microvascular disease affecting the anterior and posterior circulation. Another potential cause of the abnormal blood flow observed on TCD could be extrinsic bone compression (osteophytes and vertebral spine arthrosis). However, such a hypothesis would be less likely in our study for a number of reasons: (1) in the clinical evaluation, we excluded patients with any orthopedic problem that could have led to the vestibular symptom; and (2) we did not observe any significant variation in the blood flow through the vertebral arteries during changes in head position.

Another significant finding was the higher PI of the middle cerebral arteries (left and right) in the study group than in the control group. The increased PI in the right middle cerebral arteries may be the consequence of the higher PSV. However, such a hypothesis would not explain the higher PI we found in the left middle cerebral artery. Although we could not find any reasonable explanation for this finding in the literature, we believe that the increased PI in the left middle cerebral artery could be the result of our small sample size. Obstruction to the blood flow through the middle cerebral arteries may lead to a complex clinical syndrome characterized by contralateral hemiparesis, hemi-sensory deficit, aphasia (if the dominant hemisphere is affected) or negligence (if the nondominant hemisphere is affected), contralateral reduced visual field, dysarthria, and other cortical symptoms (3,9). The complex anatomy and the vast number of collateral vessels and territory supplied by the middle cerebral arteries may be the cause of the vast range of clinical symptoms secondary to obstruction of the blood flow. It is possible that diseases affecting the microcirculation of the middle cerebral arteries or their branches lead to some of the clinical symptoms usually attributed to occlusion of the vertebrobasilar arteries. Wong et al. (20) also demonstrated that although there is a significant anatomical distance between the trunks of the vertebrobasilar and middle cerebral arteries, infarction of their terminal branches or in borderline areas may lead to very similar clinical symptoms. Furthermore, Karnath and Dieterich (21) have demonstrated that the superior temporal gyrus (which includes the auditory cortex), the insula and the temporoparietal junction, which together integrate auditory, vestibular, proprioceptive and visual inputs, are partially supplied by the middle cerebral arteries. Additionally, Brandt et al. (22) demonstrated that patients with partial occlusion of the middle cerebral arteries might present with visual symptoms. Therefore, it is possible that pathological changes to the blood flow through the middle cerebral arteries may lead to clinical symptoms that were previously attributed solely to obstruction within the vertebrobasilar system.

In conclusion, our results demonstrate that MRA failed to detect abnormalities in patients with a clinical diagnosis of VBI. On the other hand, the PI of the basilar artery, as measured by TCD, showed high sensitivity and specificity (91%) when the PI was higher than 1.01. Considering that the PI seems to provide indirect evidence of abnormalities affecting the microvasculature, it seems that patients with a clinical diagnosis of VBI have abnormalities topographically located in the microcirculation (which are not adequately detected by MRA) rather than in larger vessels. [Table t03][Table t04]

## APPENDIX

## AUTHOR CONTRIBUTIONS

All authors were fully involved in the production of this manuscript. Lima Neto AC and Bittar RSM were the intellectual authors of the study. Lima Neto AC, Bor-Seng-Shu E, Oliveira ML, Macedo-Soares A and Topciu FR participated in the acquisition, analysis and interpretation of data. Lima Neto AC drafted the manuscript. Lima Neto AC, Bor-Seng-Shu E, Oliveira ML, Macedo-Soares A, Topciu FR and Bittar RSM critically reviewed the manuscript. All authors read the final version of the manuscript and agreed with the submission of the submitted version.

## Figures and Tables

**Table 1:** Degree of stenosis of the carotid, basilar, and vertebral arteries in volunteers from the study and control groups.

		Group			
		Study	Control	*p*-value	Comparison
MRA - right internal carotid artery	Mean	1.08	1.08		
	Median	1.00	1.00	1.000	Study = Control
	Standard deviation	0.29	0.29		
	N	12	12		
MRA - left internal carotid artery	Mean	1.17	1.08		
	Median	1.00	1.00	0.977	Study = Control
	Standard deviation	0.58	0.29		
	N	12	12		
MRA - basilar artery	Mean	0.00	0.00		
	Median	0.00	0.00	-	-
	Standard deviation	0.00	0.00		
	N	12	12		
MRA - right vertebral artery, V1	Mean	1.58	1.17		
	Median	1.00	1.00	0.630	Study = Control
	Standard deviation	1.08	0.39		
	N	12	12		
MRA - right vertebral artery, V2	Mean	1.25	1.00		
	Median	1.00	1.00	0.755	Study = Control
	Standard deviation	0.87	0.00		
	N	12	12		
MRA - right vertebral artery, V3	Mean	1.25	1.08		
	Median	1.00	1.00	0.977	Study = Control
	Standard deviation	0.87	0.29		
	N	12	12		
MRA - right vertebral artery, V4	Mean	1.33	1.00		
	Median	1.00	1.00	0.514	Study = Control
	Standard deviation	0.89	0.00		
	N	12	12		
MRA - left vertebral artery, V1	Mean	1.33	1.25		
	Median	1.00	1.00	0.977	Study = Control
	Standard deviation	1.15	0.87		
	N	12	12		
MRA - left vertebral artery, V2	Mean	1.17	1.00		
	Median	1.00	1.00	0.755	Study = Control
	Standard deviation	0.58	0.00		
	N	12	12		
MRA - left vertebral artery, V3	Mean	1.00	1.00		
	Median	1.00	1.00	1.000	Study = Control
	Standard deviation	0.00	0.00		
	N	12	12		
MRA - left vertebral artery, V4	Mean	1.25	1.25		
	Median	1.00	1.00	1.000	Study = Control
	Standard deviation	0.87	0.87		
	N	12	12		

MRA: Magnetic resonance angiography.

**Table 2:** Comparison of the TCD results between the study and control groups.

		Group			
		Study	Control	*p*-value	Comparison
TCD - left middle cerebral artery (systolic)	Mean	85.3	71.3		
	Median	87.0	73.5	0.114	Study = Control
	Standard deviation	15.1	24.0		
	N	12	12		
TCD - left middle cerebral artery (diastolic)	Mean	41.6	30.0		
	Median	41.5	30.0	0.160	Study = Control
	Standard deviation	17.2	12.2		
	N	12	12		
TCD - left middle cerebral artery (PI)	Mean	1.10	0.82		
	Median	1.08	0.82	0.005*	Study > Control
	Standard deviation	0.26	0.05		
	N	12	12		
TCD - right middle cerebral artery (systolic)	Mean	89.3	71.6		
	Median	89.5	71.8	0.012*	Study > Control
	Standard deviation	13.5	22.8		
	N	12	12		
TCD - right middle cerebral artery (diastolic)	Mean	34.2	39.5		
	Median	34.5	39.5	0.319	Study = Control
	Standard deviation	10.2	19.9		
	N	12	12		
TCD - right middle cerebral artery (PI)	Mean	1.1	0.9		
	Median	1.1	0.8	0.010*	Study > Control
	Standard deviation	0.3	0.1		
	N	12	12		
TCD - basilar artery (systolic)	Mean	59.9	62.8		
	Median	59.5	60.0	0.977	Study = Control
	Standard deviation	13.2	21.8		
	N	12	12		
TCD - basilar artery (diastolic)	Mean	22.2	33.6		
	Median	22.0	29.0	0.028*	Study < Control
	Standard deviation	6.5	19.5		
	N	12	12		
TCD - basilar artery (PI)	Mean	1.32	0.87		
	Median	1.29	0.84	<0.001*	Study > Control
	Standard deviation	0.30	0.16		
	N	12	12		

TCD: Transcranial Doppler ultrasound; PI: Pulsatility index.

* Statistically significant results.

**Table 3 t03:** 

GROUP	TCD																				
	Left middle cerebral artery			Right middle cerebral artery			Basilar artery			Left common carotid artery			Left internal carotid artery		Left vertebral artery		Right common carotid artery		Right internal carotid artery		Right vertebral artery	
VBI	PSV	EDV	PI	PSV	EDV	PI	PSV	EDV	PI	PSV	EDV	PSV	EDV	PSV	EDV	PSV	EDV	PSV	EDV	PSV	EDV
1	98	40	1.06	104	46	1.07	82	25	1.07	61.3	19.5	52.2	14	47.9	17.3	84.4	26	65.4	23.3	36.1	5.3
2	112	75	0.79	100	40	0.9	73	30	1.02	53.4	12.3	65.9	26.4	54.4	17.3	54.8	17.3	74.3	28.1	46.2	12.5
3	69	22	1.45	77	30	1.03	59	16	0.88	59.9	15.1	37.1	12.9	38.9	12.3	67.1	15.1	43.3	8.98	33.7	11.7
4	85	41	1.09	89	34	1.13	60	22	1.3	71.4	13	71.4	20.9	74	13.2	83	16.1	75.7	12.3	95.3	17.3
5	91	26	1.46	91	20	1.57	70	15	2	56.3	5.05	52.3	11.9	63.5	10.8	55.5	6.49	82.9	13.7	53.9	5.27
6	86	42	1.1	90	35	1.14	60	23	1.4	63.7	17.2	53.1	17.7	52.4	15.7	65.6	19.5	63.1	18.5	47.9	12.4
7	60	20	1.22	80	20	1.14	75	32	1.4	81.5	22.4	42.4	16.7	46.1	13.3	74.8	23.9	70.2	25.6	34.6	14.4
8	97	69	0.74	85	39	0.81	48	31	1.13	60.6	17.3	51.9	22.4	38.9	17.3	52.6	18	61.3	18.2	48.1	13.1
9	88	50	0.88	118	55	0.84	40	12	1.21	55	23.9	54.6	22.1	33.2	15.9	64.9	26	43.3	18	30.6	11.1
10	80	44	0.86	71	30	0.78	42	20	1.44	82.9	31.7	40.8	18.1	51	14.3	64.9	26	43.3	18	59.1	22.8
11	64	27	1.5	74	25	1.74	58	18	1.68	73.4	13.7	70.2	13.4	91.6	27.5	50.3	23.1	84.4	22.4	30.1	7.02
12	93	43	1	93	36	1.48	52	22	1.27	45.4	15.1	43.1	15.1	36.6	13.5	69.2	17.3	45.9	14.3	60.1	16.9

	TCD																				
	Left MCA			Right MCA			Basilar artery			Left common carotid artery			Left internal carotid artery		Left vertebral artery		Right common carotid artery		Right internal carotid artery		Right vertebral artery	
CONTROL	PSV	EDV	PI	PSV	EDV	PI	PSV	EDV	PI	PSV	EDV	PSV	EDV	PSV	EDV	PSV	EDV	PSV	EDV	PSV	EDV
1	65	28	0.93	68	50	1	51	33	0.84	74.9	19.7	49.9	19.6	36.7	7.83	83.7	23.8	44.8	16.9	44	15.1
2	70	30	0.82	71	38	0.84	63	32	0.81	69	20.7	49.5	19.6	41.6	12.2	65.9	18.4	48.6	19.3	38	12.3
3	80	53	0.79	86	45	0.79	49	23	0.84	60.6	17.3	36.8	14	29.8	11.1	55.1	19.3	53.3	19.9	43.2	22.2
4	78	39	0.75	61	30	0.75	50	20	1	103.1	32.5	85.5	33.3	32.9	9.7	77.2	15.9	65.1	22.7	30.7	6.91
5	65	30	0.81	72	39	0.85	63	34	0.75	61.3	15.1	33.4	13.7	39.3	7.09	59.9	15	28.5	12.5	30.2	6.86
6	98	40	0.75	92	40	0.79	57	20	0.87	77.7	21.7	54.5	22.3	37.4	12.3	66.3	20.9	70.6	28.6	41.2	7.85
7	74	31	0.83	71.5	40	0.86	60	20	1.33	51	15	40.4	15.3	33.8	10.8	72.1	16.6	29.3	9.61	40.4	11.5
8	110	27	0.86	116	90	0.82	128	90	0.77	72.1	20.9	72.9	26.9	66.4	26.4	66.2	18.5	48.8	19.4	39.7	12.5
9	14	1	0.77	19	3	0.74	68	47	0.84	70	25	51.6	20	66.2	16.9	80.8	20.2	59.8	26.9	59.2	15.8
10	48	22	0.84	56	28	0.94	43	26	0.84	70.7	26	32.5	14.1	30.4	6.21	50.5	18	40.4	17.3	34.1	14.6
11	80	30	0.88	75	30	1.04	60	25	0.82	53.3	14.6	77.9	29.6	66.7	23.5	46.9	14.4	98.1	25.2	36.3	14.2
12	73	29	0.8	72	41	0.85	62	33	0.77	52.3	14.2	38	16.8	43.2	14	50.5	16.8	48	20.5	35	11.7

**Table 4 t04:** 

Group	MRA										
VBI ID	Right internal carotid artery	Left internal carotid artery	Right V1 (Vertebral artery)	Right V2 (Vertebral artery)	Right V3 (Vertebral artery)	Right V4 (Vertebral artery)	Left V1 (Vertebral artery)	Left V2 (Vertebral artery)	Left V3 (Vertebral artery)	Left V4 (Vertebral artery)	Other structural findings
1	1	1	4	4	4	4	1	1	1	1	Hypoplastic right vertebral artery
2	1	1	3	1	1	1	1	1	1	1	-
3	1	1	1	1	1	1	1	1	1	1	-
4	1	1	1	1	1	1	1	1	1	1	-
5	1	1	1	1	1	1	1	1	1	1	-
6	1	1	1	1	1	1	1	1	1	1	-
7	1	1	1	1	1	1	1	1	1	1	-
8	1	1	1	1	1	1	1	1	1	1	-
9	1	1	1	1	1	2	1	1	1	1	-
10	1	1	1	1	1	1	1	1	1	1	-
11	2	3	3	1	1	1	4	3	1	4	-
12	1	1	1	1	1	1	1	1	1	1	Bilateral fetal origin of the posterior communicating artery

Control	MRA										
ID	Right internal carotid artery	Left internal carotid artery	Right V1 (Vertebral artery)	Right V2 (Vertebral artery)	Right V3 (Vertebral artery)	Right V4 (Vertebral artery)	Left V1 (Vertebral artery)	Left V2 (Vertebral artery)	Left V3 (Vertebral artery)	Left V4 (Vertebral artery)	Other structural findings
1	1	1	1	1	1	1	1	1	1	1	-
2	1	1	1	1	1	1	1	1	1	1	-
3	1	1	1	1	1	1	1	1	1	1	-
4	1	1	1	1	1	1	1	1	1	1	Persistence of the left trigeminal artery
5	1	1	2	1	2	1	1	1	1	1	-
6	2	1	1	1	1	1	1	1	1	1	Hypoplastc right vertebral artery
7	1	1	1	1	1	1	1	1	1	1	-
8	1	1	2	1	1	1	1	1	1	1	-
9	1	1	1	1	1	1	1	1	1	1	-
10	1	2	1	1	1	1	4	1	1	4	-
11	1	1	1	1	1	1	1	1	1	1	-
12	1	1	1	1	1	1	1	1	1	1	-

Table legend: TCD: Transcranial doppler. MRA: Magnetic resonance angiography. PSV: Peak systolic velocity. EDV: End diastolic velocity. PI: Pulsatility index.

## References

[1] Savitz SI, Caplan LR (2005). Vertebrobasilar disease. N Engl J Med.

[2] [No authors listed] (1990). Special report from the National Institute of Neurological Disorders and Stroke. Classification of cerebrovascular diseases III. Stroke.

[3] Grad A, Baloh RW (1989). Vertigo of vascular origin. Clinical and electronystagmographic features in 84 cases. Arch Neurol.

[4] Kim SH, Lee JS, Kwon OK, Han MK, Kim JH (2005). Prevalence study of proximal vertebral artery stenosis using high-resolution contrast-enhanced magnetic resonance angiography. Acta Radiol.

[5] Simoceli L, Bittar RM, Bottino MA, Bento RF (2003). Diagnostic approach of balance in the elderly: preliminary results. Rev Bras Otorrinolaringol.

[6] Fife TD, Baloh RW, Duckwiler GR (1994). Isolated dizziness in vertebrobasilar insufficiency: Clinical features, angiography, and follow-up. J Stroke Cerebrovasc Dis.

[7] Moubayed SP, Saliba I (2009). Vertebrobasilar insufficiency presenting as isolated positional vertigo or dizziness: a double-blind retrospective cohort study. Laryngoscope.

[8] Khan S, Cloud GC, Kerry S, Markus HS (2007). Imaging of vertebral artery stenosis: a systematic review. J Neurol Neurosurg Psychiatry.

[9] Amin-Hanjani S, Rose-Finnell L, Richardson D, Ruland S, Pandey D, Thulborn KR (2010). Vertebrobasilar Flow Evaluation and Risk of Transient Ischaemic Attack and Stroke study (VERiTAS): rationale and design. Int J Stroke.

[10] Aaslid R, Markwalder TM, Nornes H (1982). Noninvasive transcranial Doppler ultrasound recording of flow velocity in basal cerebral arteries. J Neurosurg.

[11] Nakagawa T, Yamane H, Nakai Y, Shigeta T, Takashima T (1998). Evaluation of the vertebrobasilar artery system by magnetic resonance angiography in the diagnosis of vertebrobasilar insufficiency. Acta Otolaryngol Suppl.

[12] Sloan MA, Alexandrov AV, Tegeler CH, Spencer MP, Caplan LR, Feldmann E (2004). Assessment: transcranial Doppler ultrasonography: report of the Therapeutics and Technology Assessment Subcommittee of the American Academy of Neurology. Neurology.

[13] Lempert T, Olesen J, Furman J, Waterston J, Seemungal B, Carey J (2012). Vestibular migraine: diagnostic criteria. J Vestib Res.

[14] Arnolds BJ, von Reutern GM (1986). Transcranial Doppler sonography. Examination technique and normal reference values. Ultrasound Med Biol.

[15] Namini A, Naylor M, Koenigsberg RA (2015). Vertebrobasilar Insufficiency and Stroke — A Review of Posterior Circulation Diagnostic Imaging and Endovascular Treatment Options. J Am Osteopath Coll Radiol.

[16] Nouh A, Remke J, Ruland S (2014). Ischemic posterior circulation stroke: a review of anatomy, clinical presentations, diagnosis, and current management. Front Neurol.

[17] Jacobovicz C, Timi JR, França LG, Stahlke Júnior HJ, Nakahara J (2004). Avaliação do eco-Doppler na predição da necessidade de arteriografia do território aorto-ilíaco em pacientes submetidos a revascularização arterial infra-inguinal. J Vasc Bras.

[18] Nakagawa T, Shigeta T, Takashima T, Tomiyama K (2000). Magnetic resonance angiography evaluation of basilar artery stenosis in patients with vertebrobasilar insufficiency. Eur Arch Otorhinolaryngol.

[19] Barbosa MF, Abdala N, Carrete H (2006). H, Nogueira RG, Nalli DR, Fonseca JRF, et al. Doppler transcraniano convencional em voluntários assintomáticos: variabilidade e valores de referência para parâmetros de fluxo sanguíneo. Arq Neuropsiquiatr.

[20] Wong KS, Gao S, Chan YL, Hansberg T, Lam WW, Droste DW (2002). Mechanisms of acute cerebral infarctions in patients with middle cerebral artery stenosis: a diffusion-weighted imaging and microemboli monitoring study. Ann Neurol.

[21] Karnath HO, Dieterich M (2006). Spatial neglect--a vestibular disorder? Brain.

[22] Brandt T, Dieterich M, Danek A (1994). Vestibular cortex lesions affect the perception of verticality. Ann Neurol.

